# Virtual Reconstruction and Three-Dimensional Printing of Blood Cells as a Tool in Cell Biology Education

**DOI:** 10.1371/journal.pone.0161184

**Published:** 2016-08-15

**Authors:** Ingrid Augusto, Douglas Monteiro, Wendell Girard-Dias, Thaisa Oliveira dos Santos, Simone Letícia Rosa Belmonte, Jairo Pinto de Oliveira, Helder Mauad, Marcos da Silva Pacheco, Dominik Lenz, Athelson Stefanon Bittencourt, Breno Valentim Nogueira, Jorge Roberto Lopes dos Santos, Kildare Miranda, Marco Cesar Cunegundes Guimarães

**Affiliations:** 1 Laboratório de Ultraestrutura Celular Carlos Alberto Redins, Biotecnologia, Universidade Federal do Espírito Santo, Vitória, Brazil; 2 Departamento de Fisiologia, Universidade Federal do Espírito Santo, Vitória, Brazil; 3 Departamento de Morfologia, Universidade Federal do Espírito Santo, Vitória, Brazil; 4 Ciências Farmacêuticas, Universidade de Vila Velha, Vila Velha, Brazil; 5 Museu de Ciências da Vida, Vitória, Brazil; 6 Pontifica Universidade Católica, Rio de Janeiro, Brazil; 7 Instituto de Biofísica Carlos Chagas Filho e Centro Nacional de Biologia Estrutural e Bioimagem, Universidade Federal do Rio de Janeiro, Rio de Janeiro, Brazil; University of Queensland Diamantina Institute, AUSTRALIA

## Abstract

The cell biology discipline constitutes a highly dynamic field whose concepts take a long time to be incorporated into the educational system, especially in developing countries. Amongst the main obstacles to the introduction of new cell biology concepts to students is their general lack of identification with most teaching methods. The introduction of elaborated figures, movies and animations to textbooks has given a tremendous contribution to the learning process and the search for novel teaching methods has been a central goal in cell biology education. Some specialized tools, however, are usually only available in advanced research centers or in institutions that are traditionally involved with the development of novel teaching/learning processes, and are far from becoming reality in the majority of life sciences schools. When combined with the known declining interest in science among young people, a critical scenario may result. This is especially important in the field of electron microscopy and associated techniques, methods that have greatly contributed to the current knowledge on the structure and function of different cell biology models but are rarely made accessible to most students. In this work, we propose a strategy to increase the engagement of students into the world of cell and structural biology by combining 3D electron microscopy techniques and 3D prototyping technology (3D printing) to generate 3D physical models that accurately and realistically reproduce a close-to-the native structure of the cell and serve as a tool for students and teachers outside the main centers. We introduce three strategies for 3D imaging, modeling and prototyping of cells and propose the establishment of a virtual platform where different digital models can be deposited by EM groups and subsequently downloaded and printed in different schools, universities, research centers and museums, thereby modernizing teaching of cell biology and increasing the accessibility to modern approaches in basic science.

## Introduction

The study of cells and tissues has become tightly associated with new tools since the 17th century [1–3] when biology became a breeding ground for new discoveries. The Electron Microscope (EM) emerged as a cell biology tool in the late 1930s [4] and has since enormously contributed to our understanding of cell structure and function [5, 6]. A number of EM techniques are available and currently applied in most research centers, including three-dimensional reconstruction methods [7–9].

All the progress in the cell biology field brought great possibilities to study and to understand how cells work, and how they develop and coordinate complex systems in the body. However, as science has advanced, this has become less understood and assimilated by students who might eventually contribute to the field. This gap is becoming larger and larger, demanding the introduction of novel teaching strategies that can connect scientists to students in elementary degrees of education.

The issue of declining interest in science and technology among young people has been discussed in scientific forums [10]. Among the main conclusions is the fact that negative learning experiences that occur at schools are due to the uninteresting content or poor teaching methods, which are often very detrimental to future career choices [10]. In addition, morphological sciences (anatomy, histology, e.g.) have historically been taught in the classical fashion, and new teaching approaches take too long to be implemented [10]. Information about the new discoveries in cell biology as well as the technical advances that lead to novel concepts can take a long time to reach the general public, especially in developing countries. The reasons for this include the limitation or absence of existing modern instrumentation in research centers, language barriers and lack of investment in technological development, tools, and strategies for scientific teaching [11], making science uninteresting and inaccessible for the vast majority of people.

Some authors have suggested that the role of technology in the classroom can follow two different paths: i) Learning from Technology or ii) Learning with Technology [12]. Learning from Technology involves the use of tutorials or computer-like accessories for teaching and learning purposes. In contrast, Learning with Technology focuses on using simulations and online tools for efficient organization of information [12]. Other authors have argued that the use of pedagogical models, such as teaching procedures and strategies helps translate abstract concepts into concrete knowledge [13]. Thus, technology has introduced new educational tools, thereby opening up the possibility of enhancing the quality of education and increasing the accessibility of scientific knowledge to the general public.

Although the majority of information in cell biology textbooks combines interpretations of illustrations, images, movies and animations to provide an integrated view of the molecular, biochemical, physiological and structural organization of the cell, the introduction of novel teaching tools is vital for the continuous improvement in the field. One of the reasons why students might find cell biology unappealing is the existence of illustrations and models that are non-realistic. In this regard, the use of three-dimensional (3D) illustrations has proven a good strategy to promote comprehension by replacing the flat images presented in books and thus facilitating the understanding of the morphology of organelles and their functions. As 3D prototyping technology (3D printing) is becoming less expensive and therefore more accessible, the obtaining of prototypes of “real” cells reconstructed and vectorized through 3D EM techniques may represent a novel strategy for cell biology teaching. In addition, as cell biology is predominantly a graphic discipline, it is virtually impossible for a visually impaired person to incorporate even the most basic concepts. This makes the production of 3D prototypes of cells an important route to give access to cell biology principles to an even larger public.

In this work, we obtained cell models using different 3D electron microscopy reconstruction approaches followed by 3D prototyping as alternative teaching tools to improve the engagement of students in cell biology and help professionals at all levels of education to introduce recent scientific discoveries and research devices into their classes. The 3D models werobtained from rodent blood samples analyzed by different transmission electron microscopy techniques, some of them at high resolution. Two types of leucocytes, neutrophils and monocytes, which play an important role in immune defense, were submitted to three different approaches to generate 3D models prior to prototyping: (1) Acquisition of a single projected image from a thin section in the Transmission Electron Microscope (TEM) followed by artificial vectorization, (2) 3D reconstruction from serial sections using a classical (low resolution) approach (available in most electron microscopy labs) and (3) 3D reconstruction at high resolution by Serial Electron Tomography [14]. All vectorized models obtained were then printed using a 3D prototyping machine. Differing from traditional cell models that are currently used in biology teaching, which usually illustrate a rounded cell with a static and regular membrane, the models generated represent cells that are morphologically close to that of contemporary scientific knowledge. We suggest the establishment of a virtual platform where different 3D data from cells and molecules already obtained by different groups could be deposited so that virtual and physical models can be obtained and made available at different schools. Whether or not these strategies improve the learning processes by students is currently under investigation by our group.

## Material and Methods

### Ethics statement

This study was approved by the Ethics Committee for Animal Experimentation of the Health Sciences Centre of the Federal University of Espírito Santo, under the Protocol number 033/2014 according to the Brazilian federal law (11.794/2008, Decreto n° 6.899/2009), which is based on the “Guide for the Care and Use of Laboratory Animals” prepared by the National Academy of Sciences, USA, and the “Australian Code of Practice for Care and Use of Animal for Scientific Purpose.” All animals received humane care in compliance with the above-mentioned guides.

### Animals

The Central Animal House of the Federal University of Espírito Santo provided Wistar male rats weighing 150-180g. The rats were deeply anesthetized with ketamine (70 mg.kg-1, i.p.) and xylazine (10 mg.kg-1, i.p., Bayer, Brazil). The rats were cannulated to collect blood for leukocytes assay and euthanized by opening the thorax and removing the heart. During this study the animals were maintained in a 12-h light/dark cycle in a controlled-temperature room (22±1°C), 50±5% humidity, standard rat chow and water *ad libitum*.

### Cell isolation

In total, 10 ml of venous blood was extracted from the left ventricle of two control rats. The blood was collected in two 5 ml EDTA tubes and submitted to a Ficoll-Hypaque density gradient to separate the cells with the least possible damage. The blood was diluted 1:1 with PBS and mixed with 3 ml of Ficoll solution in another tube. The samples were centrifuged at 1400 rpm for 10 minutes, forming a white clot of leucocytes in the middle of the tube. After collection, they were washed with PBS. The samples were again centrifuged following the previous steps. The final step required washing and centrifugation of the samples at 1000 rpm for 7 minutes.

### Cell preparation

Once the leucocyte pellet formed, the cells were fixed in 2.5% glutaraldehyde (2 hours), followed by post-fixation in 1% osmium tetroxide (30 minutes in a dark room). The cells were then washed once with 0.1 M cacodylate buffer and twice with distilled water for 10 minutes each. The following dehydration promoted the replacement of the aqueous portion of the cell with acetone, which later helped resin efficiently penetrate into the cell. The substitution of water was performed using an acetone gradient bath, at 10 minutes each at the following concentrations: 50%, 70%, 90% and 100%. The last gradient was repeated 3 times. All samples were embedded in epoxy resin (Epon 812) and acetone at 1:1 for 12 hours, followed by epoxy embedding for another 12 hours. The samples were then transferred to silicon molds and covered with pure epoxy. The blocks were heated at 60°C for 48–72 hours to allow polymerization.

### 3D Reconstruction

#### Vectorization of a single image

Thin sections of the samples prepared as above were obtained using an ultramicrotome (PT-XL Power Tomes, RMC, USA). Sections were stained with uranyl acetate (5%) for 30 minutes followed by lead citrate (1%) for 5 minutes and examined under a transmission electron microscope (JEM 1400, JEOL, Inc., USA) operation at 80 kV. Image of a neutrophil in a single ultrathin section (70 nm thick) acquired with a CCD CAMERA (2k x 2k) (Gatan Orius SC200, model 830, Gatan USA) were used to produce a 3D model by artificially increasing the height (z scale) of each structure previously segmented. The artificial 3D volume was created with the use of the Rhinoceros software (Robert McNeel & Associates, Seattle).

#### Serial sectioning

3D reconstruction from serial sections is defined by the acquisition and reassembly of images from different profiles of the same cell in sequential sections [7, 15]. A ribbon of 30 serial slices (70 nm thick) was obtained with an ultramicrotome (PT-XL Power Tomes, RMC, USA). The block side facing the knife was coated with a thin layer of glue to prevent dissociation of the sections. After sectioning, a slot grid was used to collect the ribbon and placed onto a Formvar layer and air-dried. The sample was stained as before and examined under a Transmission Electron Microscope (JEM 1400, JEOL, Inc., USA) operation at 80 kV. Images of profiles of a neutrophil in consecutive sections were obtained and aligned with the use of AMIRA (FEI Company, USA). Segmentation was performed in the whole dataset and the profiles of the main structures of the cell rendered automatically, producing a virtual 3D model as shown below.

#### Serial electron tomography

Electron tomography consists of acquiring a tilt series of projected images in the electron microscope, followed by a number of image processing and digital reconstruction steps that generate a 3D volume. Here, a 200 kV Transmission Electron Microscope (Tecnai G2, FEI Company, Eindhoven) equipped with a 4k x 4k CCD camera (Eagle, FEI Company, Eindhoven) was used to record approx. 25 consecutive tomograms of a monocyte from a series of 200 nm thick sections. Tilt series from -65° to +65° with an angular increment of 1° were used to acquire all tomograms. Alignments were applied using fiducial markers and weighted back projections were done with the use of an IMOD software package (University of Colorado, USA). For segmentation and data display, AMIRA (FEI Company, USA) and IMOD [16] were used.

#### 3D printing

The vectors used on the process of 3D printing were exported from the 3D models as VRML language (Virtual Reality Modeling Language) and rendered on Rhinoceros 3D (Robert McNeel & Associates, Seattle, Trial License). Repetier-Host for FELIXPrinters (FELIXrobotics bv, Netherlands) was used to print on a FELIX 3.0 Dual Extruder (Felixprinters, Netherlands) 3D printer. Both extruders were used, the first one using a PLA (Polylactic acid) filament for cell structures and the second one a PVA (Polyvinyl alcohol) as support material. The physical modelling process was conducted by resolving the layers of composite powder agglutinated with liquid binder. The 3D geometry was achieved by agglutinating each layer and gradually lowering the base.

## Results

**Fig 1:**
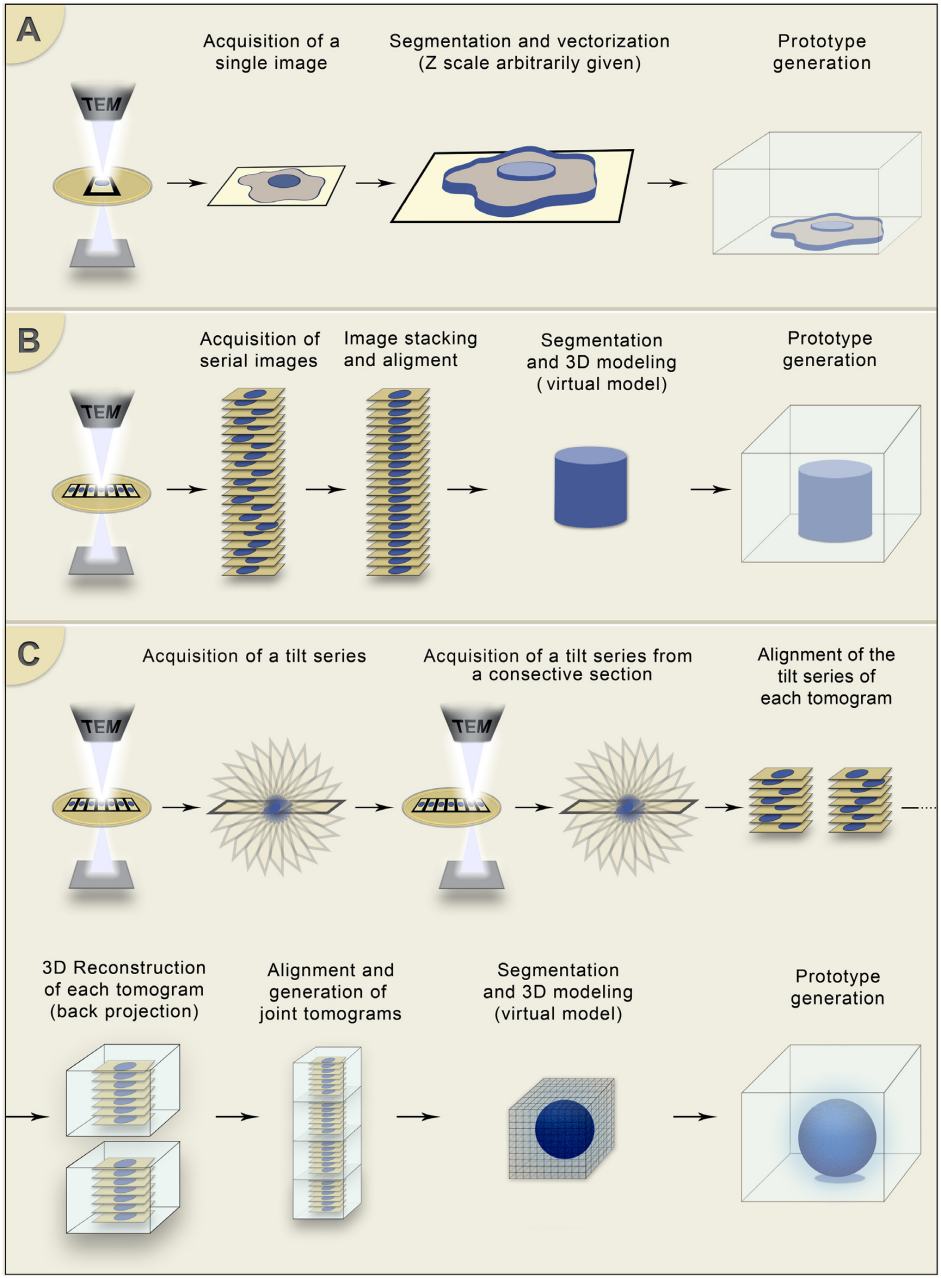
Schematic representation of the techniques used to obtain the 3D models and prototypes. (A) Vectorization from a single projected image obtained by conventional transmission electron microscopy. (B) Generation of a 3D model by serial-section transmission electron microscopy. (C) Generation of 3D model by serial electron tomography. All 3D models were converted to the appropriate language (readable by 3D printing software) and used to generate a prototype.

Prototypes of cell profiles and the whole cell volumes were obtained by using different transmission electron microscopy applications. Fig 1 summarizes the main strategies used here to produce virtual and physical models (3D prototypes) of cells, from analysis of single thin sections (Fig 1A) to serial electron tomography (Fig 1C). Results obtained with each technique are detailed below.

### 3D prototyping of models obtained from thin sections

**Fig 2:**
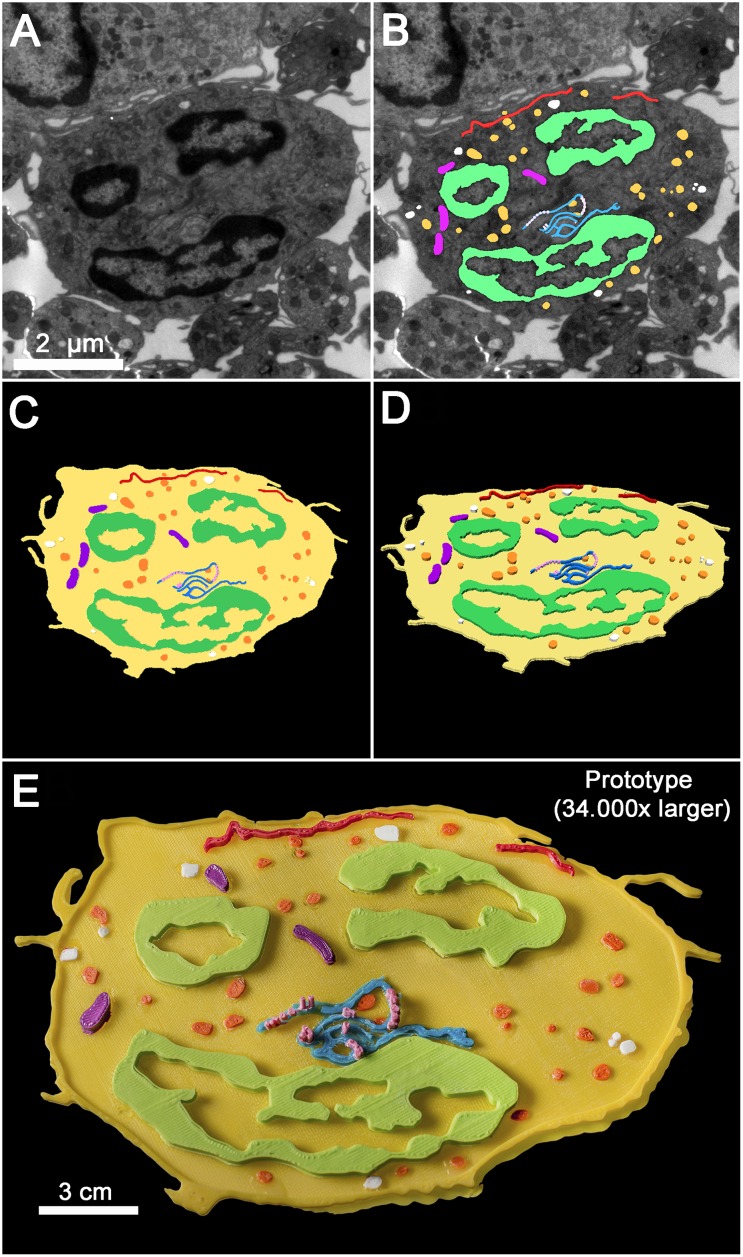
3D representation of the neutrophil used on 3D printing. (A) Transmission electron microscopy of a thin section of a neutrophil. (B) Segmentation of different internal structures, (C) model showing the main structures segmented form the previous images and (D) virtual model resulted from image vectorization (artificial increase in the z scale). Primary granules (orange), secondary granules (white), nucleus (green), rough endoplasmic reticulum (blue), endoplasmic reticulum (red), mitochondria (purple). (E) Printed prototype of the neutrophil 34,000 times larger than the virtual model.

Analysis of ultrathin sections of leucocytes by TEM produced projected images that revealed profiles of neutrophil cell surface and most intracellular structures (plasma membrane, nuclei, endoplasmic reticulum, mitochondria, primary granules, secondary granules, etc), providing details on the ultrastructural organization of this cell type, as expected (Fig 2A). Digital segmentation (manual delineation) of each structure of interest generated an equal number of objects—flat colored representations of the membranes and organelles (Fig 2B and 2C)—that were then processed to gain an artificial surface volume (arbitrary increase of the z scale) (Fig 2D). This was carried out through a vectorization strategy using the Rhinoceros platform. The virtual 3D representation of the cell profile (3D model) was then converted to the appropriate language readable by a 3D prototyping machine (3D printer) and a physical prototype containing a detailed distribution of its surface as well as internal structures was generated (Fig 2E). The prototype (20 cm wide) was approximately 34,000 times larger than the original cell and highlighted the main structures present in the cell. To make it available to every interested scholar, a file of the model containing the vectorized coordinates required for 3D printing was uploaded and is available to download (S1 File).

### 3D prototyping of models obtained from serial sections

**Fig 3:**
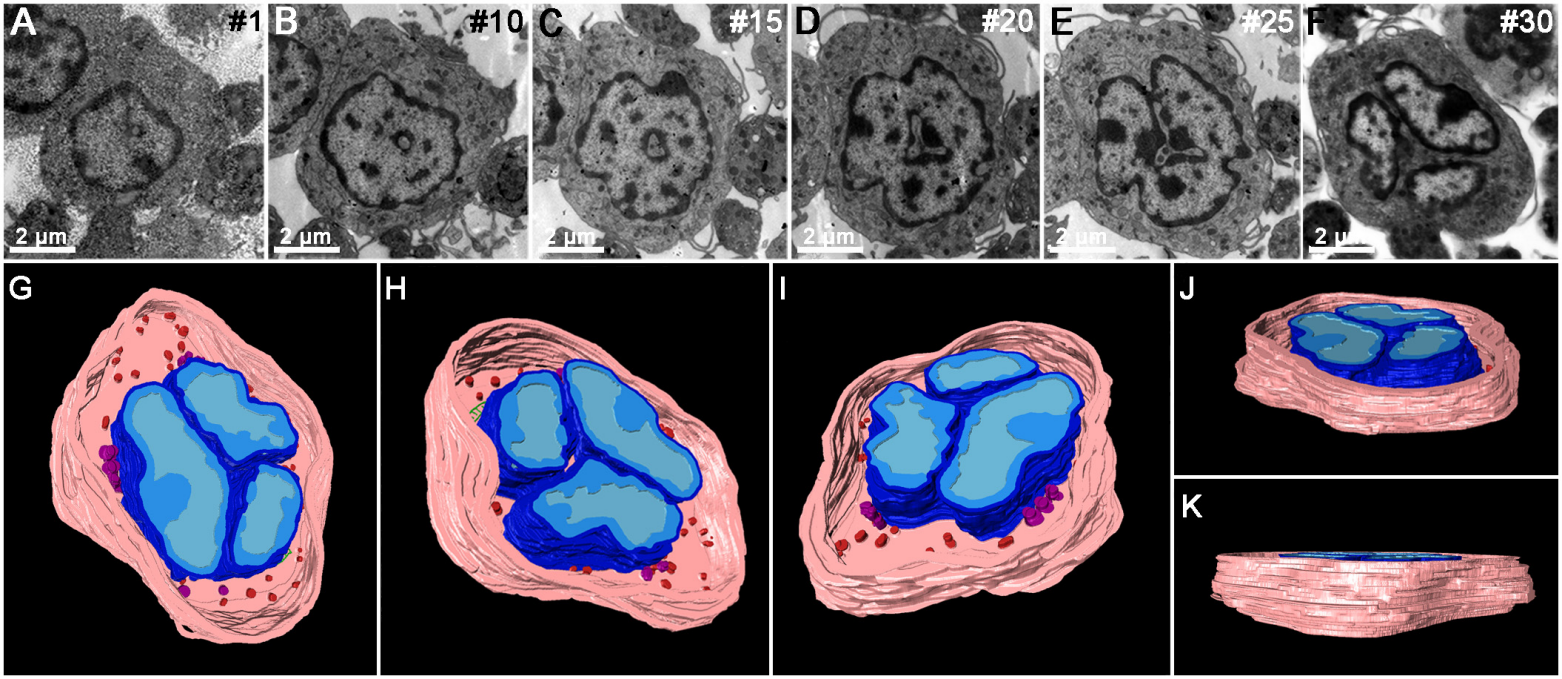
3D reconstruction of the whole volume of a neutrophil by serial section. (A-F) Obtainment of serial images from 70 nm-thick serial sections of a neutrophil. (G-K) 3D model showing the cell nucleus (blue) with heterochromatin (dark blue) and euchromatin (light blue), plasma membrane (light pink) and primary (red) and secondary granules (purple).

In order to reconstruct the larger volumes of cells, two main strategies were used: (1) 3D reconstruction from serial sections and (2) 3D reconstruction from serial tomography. As one of the aims of the present work is to reach the highest possible number of groups, which may have access to equipment with different levels of sophistication, we started with a classical method that can be applied in any EM lab. Series of approx. 30 thin sections were obtained and collected in formvar-coated slot grids. Images of each profile of the neutrophil in different sections were individually acquired (Fig 3A–3F), the stack of images aligned and some of the structures segmented. Rendering of the different segmented profiles resulted in a 3D virtual model (Fig 3G–3K) that reveals the typical internal structures of a neutrophil and exemplifies how dynamically organized they are along the cell volume. Once obtained, this vectorized model is available for 3D printing. To make this model easily comparable with the model obtained by serial tomography, only the nuclei, the surface membrane and a few organelles are shown. A file of the vectorized model is also available for download (S2 File).

### 3D prototyping of models obtained from serial tomography

**Fig 4:**
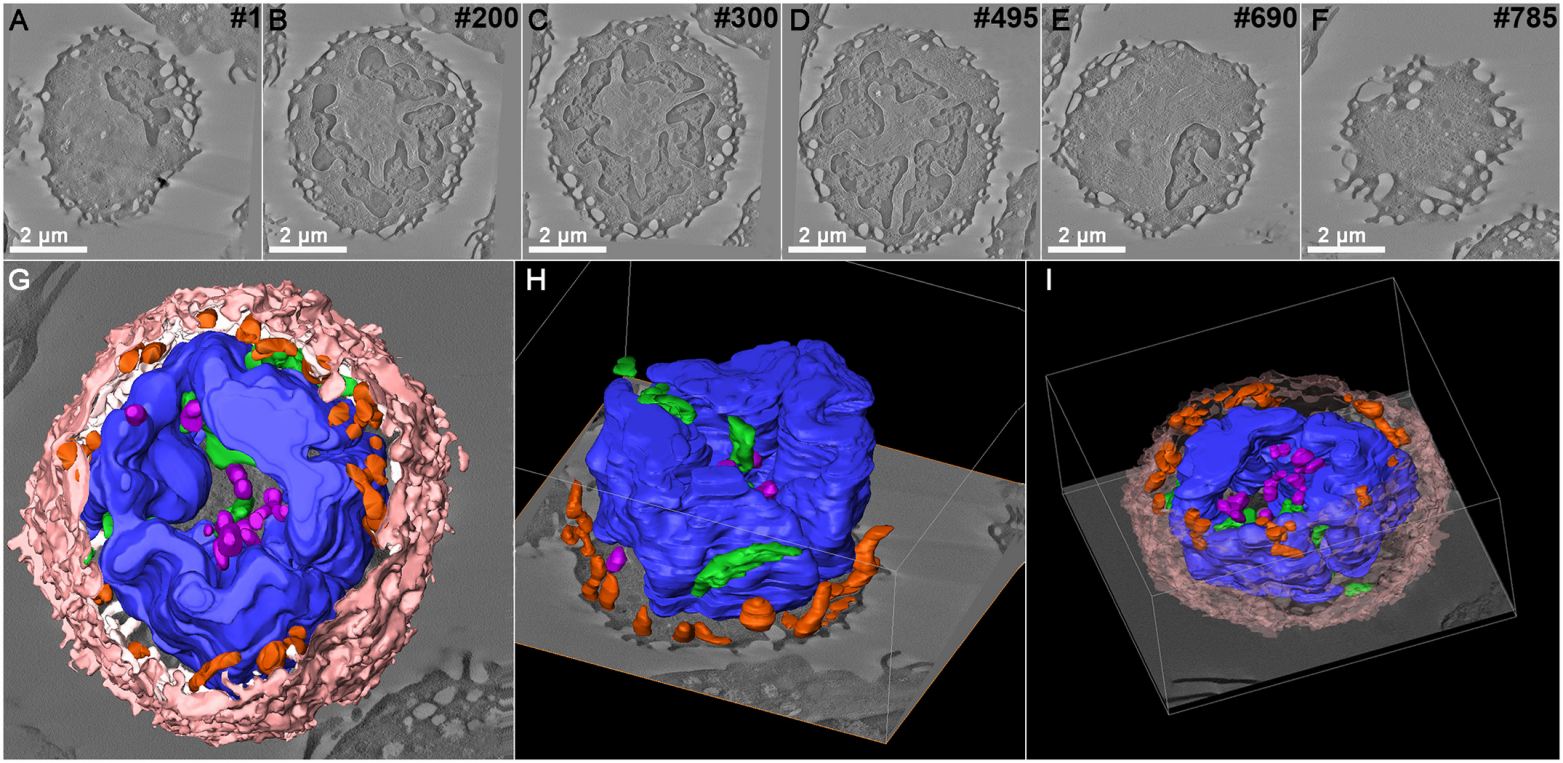
3D representation of the monocyte used on 3D printing. (A-F) Virtual sections from a serial tomogram of a monocyte obtained from serial electron tomography. Bar 2 μm. (G-I) 3D representation of the model, showing the cell nucleus (blue), plasma membrane (light pink), mitochondria (green), lysosomes (purple) and phagosomes (Orange).

**Fig 5:**
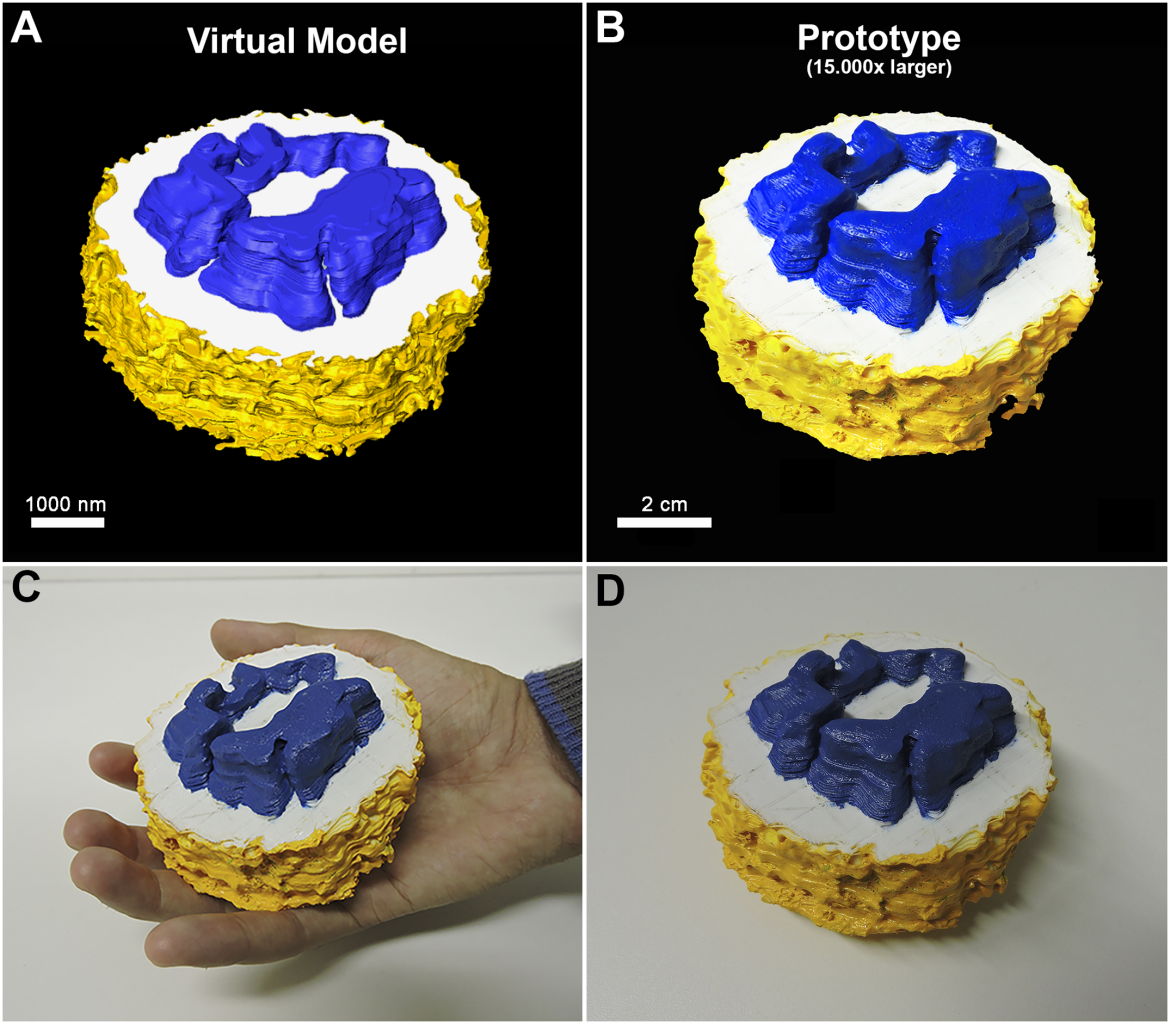
3D prototypes obtained form a virtual model of a monocyte. (A) Virtual model of a monocyte generated by serial electron tomography. (B) The resulting in a prototype 15.000 times larger than the virtual model. (C) Printed prototype being manipulated by a student and (D) on a table.

As mentioned before, 3D models of a large volume of a cell were obtained by Serial Section Electron Tomography, considered a low resolution technique, but operationally available to the most EM labs. To improve the resolution of the reconstructed images and volumes, 3D reconstructions was carried out through electron tomography, one of the current modern techniques that has been applied in most 3D EM labs. As in all TEM based techniques, there is a limitation in the section volume (thickness) that can be imaged by this technique. To reconstruct larger volumes (the whole thickness of a cell), we combined the serial section strategy with tomography and obtained approx. 25 tomograms of a monocyte from 250 nm consecutive sections, as described above (Fig 4A–4F). Joint tomograms containing high-resolution information from both intracellular and surface structures were obtained (Fig 4G–4I). 3D modelling and segmentation resulted in a 3D virtual model that showed the 3D organization of typical structures such as the projections of the plasma membrane, the complex structure of the nucleus, peripheral phagosomes, elongated lysosomes, and mitochondria mainly located at the central portion of the cell (Fig 4G–4I and S1 Movie). These are characteristics typical of this cell type, as this is a precursor of macrophages. To compare with the information obtained by serial section EM, the virtual model was simplified and used to generate a prototype (Fig 5 and S1 Movie). Files of the vectorized model are also available for download (S3 File).

## Discussion

In this study, three-dimensional electron microscopy techniques associated with emerging three-dimensional prototyping technologies were used to generate virtual and physical reconstructions of blood cells. Three different approaches to generate 3D models are proposed here to be used as a complementary tool in cell biology teaching. The central idea behind this proposition is the current possibility of using hundreds of published (and unpublished) models generated through different EM techniques, print them and present them to the students as an additional tool to provide a more realistic perception of a “real” cell model.

Introduction of didactic drawings and graphic animations in texts books and websites has spectacularly expanded our communication capability beyond the models of the “fried egg” type usually found in old textbooks. These didactic drawings have reached a great number of students studying in different disciplines and degrees of education. Among the reasons for such success is the simplicity of the language used, which has been shown to sound more credible than when erudite vernacular terms are used [17], and the identification of youth with the tools used in the learning processes, usually a computer-based approach (mobile apps, movies, etc). In this regard, the introduction of a strategy that makes use of the generation of virtual models and 3D printing, technologies that have gained a lot of attention among young students, may represent a step forward in the teaching process. A remarkable potential of these techniques is the instantaneous engagement of the students (as actually occurred during the experimental work of this project). In addition, introduction of models and printed prototypes that present a higher similarity to the “real” cell structure as observed in the microscope may also shorten the gap between discovering the information through experimental work and learning it in the textbook.

The continuous search for novel complementary strategies to teach cell biology requires a constant update on the repertoire of techniques used to produce schematic representations and models, including other 3D EM techniques. Here we used three TEM-based 3D reconstruction techniques: (1) vectorization of models obtained from thin sections (discussed below), (2) classic serial sectioning EM and (3) serial electron tomography. A number of SEM-based methods could be used as well to provide virtual and printed models. These comprise Serial Block-Face Scanning Electron Microscopy (SBF-SEM) [18], Focused Ion Beam-Scanning Electron Microscopy (FIB-SEM) [19], Serial Section Scanning Electron Microscopy (SSEM) [20] and Array Tomography [21]. All of these techniques can provide information of larger volumes, although with lower resolution than in electron tomography. As in TEM tomography, these approaches require expensive and sophisticated equipment that are not yet available in most laboratories, although published models obtained in such labs could also be made available for downloading. Improved cell or tissue preservation with the use of cryotechniques could also improve the quality of information so that the models resemble the cell in a more reliable close-to-the-native state [22].

Regarding the vectorization and 3D printing of thin sections, we believe that this may have potential when applied in teaching cell biology to visually impaired students. Our model provides a unique chance for them to understand the intracellular environment (as opposed to surface details if the whole cells is printed) as visualized by scientists by touching prototypes obtained from artificially vectorized models. This is an innovation of high applicability that can greatly benefit visually impaired people since any graphic object with vectorized coordinates can be printed (not only cells) and the rigid material touched to mentally reconstruct the image.

Developed in 1980/1990 [23, 24], the 3D printing technology has since been applied in automotive, film and pharmaceutical industries, for the production of jewelry, prostheses for humans and other animals, and increasingly in basic research [23–26]. Although most schools may not easily access cell biology labs or electron microscopy facilities, the cost of 3D printers is becoming less and less prohibitive. Based on this scenario it is therefore entirely conceivable that teachers and students, besides studying printed cell models, could also print virtual models provided by different 3D EM groups.

In summary, our data reveal that reconstruction via 3D cellular modeling is able to bring together science, technology and basic education, thereby modernizing science teaching to engage both sighted and visually impaired students by applying 3D printing.

## Supporting Information

S1 File File of a vectorized model from an ultrathin section of a neutrophil for 3D printing. (3DS)

S2 File File of a vectorized model obtained from serial sectioning for 3D printing. (3DS)

S3 File File of a vectorized model obtained from serial electron tomography for 3D printing. (3DS)

S1 Movie Movie showing virtual sections through tomograms obtained from serial electron tomography of a monocyte. The virtual model generated is animated in sequences of images to show different angles. As this sequence fades, a sequence of images of the prototype also at different angles is revealed.
(M4V)
